# The Association of Serum Uric Acid with Risk of Obstructive Sleep Apnea: The Korean National Health and Nutrition Examination Survey 2019–2021

**DOI:** 10.3390/jpm14050532

**Published:** 2024-05-16

**Authors:** Su-Lim Park, Jihye Lim, Ji-Ho Lee

**Affiliations:** 1 Department of Medicine, Yonsei University Wonju College of Medicine, Wonju 26426, Republic of Korea; psl8058@yonsei.ac.kr; 2 Department of Medical Informatics and Biostatistics, Yonsei University Wonju College of Medicine, Wonju 26426, Republic of Korea; jihye8082@yonsei.ac.kr; 3 Department of Internal Medicine, Yonsei University Wonju College of Medicine, Wonju 26426, Republic of Korea

**Keywords:** obstructive sleep apnea, uric acid, STOP-Bang, prevalence, risk factor

## Abstract

Upper airway collapse and apneas in obstructive sleep apnea (OSA) induce intermittent hypercapnia and hypoxia, eventually contributing to excessive uric acid production. This study aimed to evaluate the association between hyperuricemia and OSA in the general population via analysis of the eighth KNHANES dataset (2019–2021). OSA risk was identified via the STOP-Bang questionnaire, with a score ≥3 indicating high risk. Among 11,981 total participants, 4572 (38.2%) had a high OSA risk. Participants with a high OSA risk had higher uric acid levels compared to those with a low risk (5.5 ± 1.4 mg/dL vs. 4.8 ± 1.2 mg/dL, *p* < 0.001). Serum uric acid levels were positively correlated with STOP-Bang score (r: 0.317, *p* < 0.001). Multivariate analysis revealed that hyperuricemia was associated with a high OSA risk after adjusting for confounders (odds ratio: 1.30, 95%CI: 1.11–1.53, *p* = 0.001). Therefore, serum uric acid levels are significantly higher in those with a high OSA risk and correlate with the risk of OSA. Further, hyperuricemia is an independently associated risk factor for high OSA risk. More research is warranted to evaluate the long-term clinical outcomes of hyperuricemia in OSA and to determine whether treatment targeting hyperuricemia is effective in the clinical course of OSA.

## 1. Introduction

Obstructive sleep apnea (OSA) is a clinical condition characterized by episodes of complete or partial collapse of the upper airway during sleep, leading to breathing cessation (apnea) or significant reduction in airflow (hypopnea). These episodes result in reduced oxygen saturation in the blood and frequent awakening to resume breathing, significantly disrupting sleep quality [1]. OSA affects a broad spectrum of the population with varying prevalence rates [2]. The prevalence of OSA, as measured using polysomnography in sample populations, was 4% in Hongkong and 26% in the United States [3,4]. Factors contributing to the increased prevalence include aging populations and rising obesity rates, which are linked to the anatomical and functional changes in the airway that predispose to airway obstructions [5]. Individuals with OSA may present a range of symptoms, from none at all to severe sleep disruption, and often suffer from multiple health consequences [6]. These include cardiovascular issues such as hypertension and increased risks of heart disease, metabolic syndrome, renal dysfunction, chronic obstructive pulmonary disease, and various neuropsychiatric disorders including depression, influenced by the severity of OSA [7]. Research highlights that OSA not only significantly increases the risk of stroke but also poses heightened risks for both fatal and non-fatal cardiovascular events in patients with severe, untreated forms of the condition [8]. Furthermore, the disruption in sleep quality and the resultant daytime sleepiness significantly impacts cognitive functions and increase the likelihood of vehicular accidents, with OSA patients being up to seven times more likely to be involved in car accidents compared to those without the disorder [9]. As such, OSA significantly diminishes quality of life and presents substantial challenges to public health, necessitating effective management and treatment strategies to mitigate its widespread impact.

Despite the growing recognition of obstructive sleep apnea as a significant health is-sue, a large number of patients remain unaware of their condition. Studies estimate that 93% of women and 82% of men with moderate-to-severe forms of sleep apnea syndrome have not been clinically diagnosed [10]. This underdiagnosis is largely due to the challenges associated with the primary diagnostic tool, night-time polysomnography. This gold standard for diagnosis, while effective, is also known for being cumbersome, expensive, and time-consuming, contributing significantly to the high rate of undiagnosed cases [11]. Recognizing the critical need for more accessible diagnostic tools, the eighth Korean National Health and Nutrition Examination Survey (KNHANES, 2019–2021) introduced a screening questionnaire specifically for OSA for the first time. This marked a pivotal moment in addressing the underdiagnosis of this condition. KNHANES is renowned for its extensive sample sizes, standardized data collection methodologies, and stringent quality control measures. These features are critical as they enable researchers to gather data from a wide demographic, ensuring that the findings are robust and reliable.

Hyperuricemia refers to elevated levels of uric acid in the blood. Hyperuricemia is a well-known major risk factor for gout but is also a risk factor for chronic kidney disease (CKD) [12]. Elevated serum uric acid levels are associated with renal dysfunction, as excessive uric acid can lead to urate crystal deposition in the kidneys, which in turn can cause inflammation and damage [12]. Hyperuricemia has been linked to increased cardiovascular disease risk. This connection is due in part to the role of uric acid in inducing oxidative stress and inflammation, factors that contribute to atherosclerosis and other cardiovascular pathologies [13]. The prevalence of hyperuricemia is approximately 20% in Western countries [14]. Asian countries report a lower prevalence of hyperuricemia compared to Western countries. The prevalence of hyperuricemia was 13.3% in a meta-analysis of studies reported between 2000 and 2014 in China and 11.4% in the 2016 Korean National Health and Nutrition Examination Survey (KNHANES) [15,16]. As hyperuricemia is a comparatively common disorder, studies evaluating its relationship with various comorbidities have also been reported [17,18].

While each condition has been extensively studied in isolation, emerging evidence suggests a complex interplay between OSA and elevated uric acid levels. Understanding the association between OSA and high uric acid levels will provide a deeper insight into disease prognosis and treatment strategies for OSA. To the best of our knowledge, no study has examined the association of hyperuricemia with OSA in the Korean population. Therefore, this study aimed to evaluate the association between hyperuricemia and the risk of OSA using a validated questionnaire.

## 2. Materials and Methods

### 2.1. Study Population

**Figure 1:**
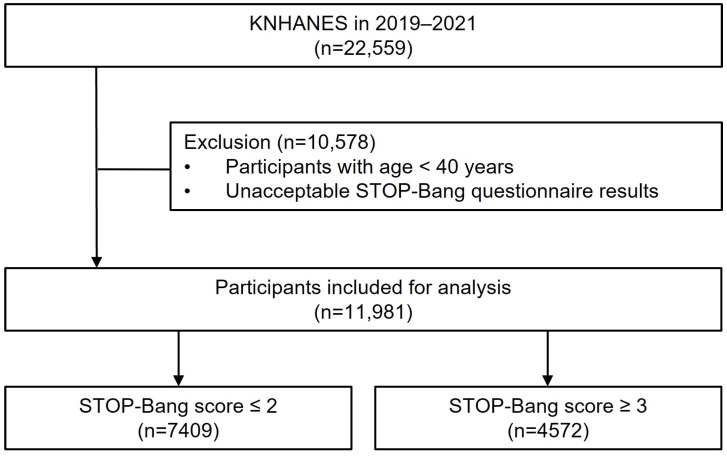
Flowchart of patient selection.

This study analyzed the dataset of the eighth KNHANES (2019–2021). The KNHANES has been conducted by the Korean Centers for Disease Control and Prevention (KCDC) since 1998. The KNHANES employs a probability sampling method to evaluate the health and nutritional conditions of a reliable national representation of the noninstitutionalized civilian population in Korea. The study encompasses three elements: a health interview, a health examination, and a nutrition survey. Proficient interviewers and laboratory technicians execute the data collection process. The details of the KNHANES are provided online (https://knhanes.kdca.go.kr/knhanes/eng/index.do, accessed on 18 October 2023). Of the 22,529 total participants during 2019–2021, we excluded 10,578 participants who were of a young age (<40 years) and had missing data for the variables of interest. Overall, 11,981 subjects were included in this study (Figure 1).

The study protocol was conducted following approval by the Institutional Review Board at the KCDC. All participants were fully informed of the protocol and provided written informed consent.

### 2.2. Data Collection

Demographic and socioeconomic data, including age, sex, height, body weight, and household income, were collected. The presence of relevant comorbidities, such as hypertension, hyperlipidemia, stroke, myocardial infarction or angina, diabetes, chronic kidney disease, and gout, were defined based on physician diagnostic history. Body mass index (BMI) was calculated as body weight divided by height squared (kg/m^2^). The categorization of smoking status involved labeling individuals as “never” smokers if they had never smoked or had a history of consuming fewer than 100 cigarettes. Participants were designated as “former” smokers if they had abstained from smoking for a minimum of 6 months but had previously smoked more than 100 cigarettes. The classification of “current” smokers encompassed individuals actively smoking during the study or those who had quit within the preceding 6 months. The household income was divided into 4 groups of quartiles according to 25%, 50%, and 75% (Q1–Q4) of the population. Higher quartiles indicated a higher-income group.

### 2.3. Study Parameters

The snoring (“Do you snore loudly?”), tiredness (“Do you often feel tired, fatigued, or sleepy during the daytime?”), observed apnea (“Has anyone observed you stop breathing during sleep?”), high BP (“Do you have high blood pressure?”), BMI, age, neck circumference, and male gender (STOP-Bang) questionnaire was used to screen for OSA [19]. The BMI cut-off value was set as 30 kg/m^2^ instead of 35 kg/m^2^ following criteria for Asian obesity classification in a previous study [20]. Each question was answered as “yes” and “no” and scored as 1 and 0, respectively. The total scores ranged from 0 to 8. A total score of 3 or more positive responses was regarded as high risk for OSA.

Serum uric acid was measured by the calorimetry method using a Hitachi Automatic Analyzer 7600–210 (Hitachi Medical Corporation, Tokyo, Japan). Hyperuricemia was defined as a serum uric acid level ≥7.0 mg/dL in men and ≥6.0 mg/dL in women [21].

### 2.4. Statistical Analysis

To achieve fair national estimates of the overall Korean adult population, the KNHANES adjusted sampling weights to accommodate a multifaceted sample design. This involved applying sampling techniques to distinct strata, including primary sampling units and households. After adopting the weight values, statistical analysis was conducted. Descriptive statistics to compare baseline characteristics between low risk and high risk of OSA were described using Student’s *t*-test for continuous variables and the chi-squared test for categorical variables. The Spearman correlation coefficient (r) was used to identify the correlation between uric acid levels and STOP-Bang scores. Binary logistic regression was conducted to identify the risk factors associated with a high risk of OSA, which served as the dependent variable. Logistic regression analysis included diverse demographic and clinical variables as baseline characteristics. Significant variables identified in the univariate analysis were included in the multivariate analysis. Odds ratios (ORs) were analyzed to identify associated factors with high risk of OSA. We additionally analyzed whether the STOP-Bang score could be used to investigate the association between OSA risk and uric acid levels. A cohort with propensity score matching of variables, including B (BMI), A (age), N (neck circumference) and G (male gender), were made between low risk of OSA (STOP-Bang ≤ 2) and high risk of OSA (STOP-Bang ≥ 3). Logistic regression analysis was conducted to investigate the association between the STOP (snoring, tiredness, observed apnea, and hypertension) and uric acid levels. All statistical analyses were performed using SAS version 9.4 (SAS Institute Inc., Cary, NC, USA). Statistical significance was set at *p* < 0.05.

## 3. Results

Of the 11,981 total participants, 7409 (61.8%) and 4572 (38.2%) were classified as having a low risk (STOP-Bang score ≤ 2) and high risk (STOP-Bang score ≥ 3) of OSA, respectively (Table 1). The mean age and proportion of men was significantly higher in the high-risk group when compared with the low-risk group (age: 63.4 ± 10.6 years vs. 57.7 ± 11.8 years, *p* < 0.001; men: 71.0% vs. 26.5%, *p* < 0.001). The high OSA risk group had a greater proportion of former and current smokers (60.9% vs. 27.7%, *p* < 0.001) and a higher proportion of BMI ≥ 30 (11.6% vs. 1.7%, *p* < 0.001). Relevant comorbidities were more prevalent in the high-risk group, whereas household income was significantly lower in this group than in the group with a low risk (all *p* < 0.001).

**Figure 2:**
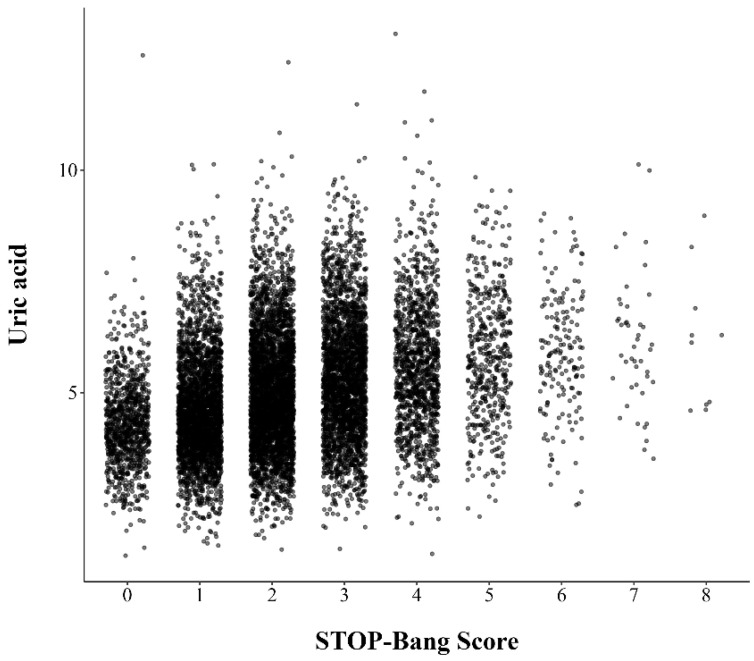
Jitter plot representing distribution of serum uric acid levels at single point of STOP-Bang score.

The mean serum uric acid level for all participants was 5.1 ± 1.4 mg/dL. Subjects with a high risk of OSA had higher uric acid levels compared to those with a low risk (5.5 ± 1.4 mg/dL vs. 4.8 ± 1.2 mg/dL, *p* < 0.001). The distribution of the uric acid levels assigned to each STOP-Bang score is depicted in a jitter plot (Figure 2). Serum uric acid levels were positively correlated with STOP-Bang score (r: 0.317, *p* < 0.001).

In a univariate analysis, old age, male sex, being a former and current smoker, high BMI, and the presence of comorbidities were significantly associated with a high risk of OSA, whereas higher household income was a protective factor (all *p* < 0.05) (Table 2). Hyperuricemia was associated with a high risk of OSA in univariate analysis (OR: 2.01, 95% CI: 1.78–2.27, *p* < 0.001) and multivariate analysis after adjusting for confounders (OR: 1.30, 95% CI: 1.11–1.53, *p* = 0.001).

A cohort of low OSA risk and high OSA risk was constructed using propensity score matching. The standardized mean difference was within the acceptance limit of 0.5 after matching (Table 3). All variables of STOP (snoring, tiredness, observed apnea, and hypertension) and uric acid levels were significant in a univariate analysis. Uric acid levels were significantly associated with high OSA risk after adjusting for other independent variables (OR: 1.36, 95% CI: 1.28–1.44, *p* < 0.001) (Table 4).

## 4. Discussion

The results of this study showed that a substantial proportion of the general population was at high risk of OSA (STOP-Bang score ≥ 3). Subjects with a high risk of OSA had a higher prevalence of relevant comorbidities and low household income. Serum uric acid levels were significantly higher in those with a high risk of OSA compared to those with a low risk. There was a positive correlation between uric acid levels and STOP-Bang score. Hyperuricemia was independently associated with a high risk of OSA in a logistic regression analysis.

OSA is intrinsically linked to the elevation of uric acid levels, primarily through mechanisms involving intermittent hypoxia and hypercapnia caused by repeated episodes of upper airway collapse during sleep. These episodes lead to significant oxygen desaturation, frequent arousals, and a subsequent increase in blood CO_2_ levels [22]. The resultant hypoxia and reoxygenation cycles foster the production of reactive oxygen species, which are critical mediators of oxidative stress [23]. This oxidative stress impairs mitochondrial function, crucial for cellular energy production, hindering the synthesis of adenosine triphosphate (ATP). The deficiency in oxygen supply affects mitochondrial autophagy and the normal cellular degradation processes, which further disrupt ATP production. When ATP synthesis is compromised, the breakdown of ATP to adenosine diphosphate (ADP) and adenosine monophosphate (AMP) is accelerated [24]. This cascade leads to the release of purine degradation products, such as adenosine, hypoxanthine, and xanthine, which are enzymatically broken down into uric acid, the end product of purine metabolism. Furthermore, the continuous state of hypoxia characteristic of OSA promotes poly (adenosine diphosphate-ribose) polymerase activation, a response to DNA damage, which exacerbates ATP depletion and elevates ADP and AMP levels, thereby increasing uric acid production [25]. This mechanistic pathway elucidates how disturbances in cellular and mitochondrial function in OSA patients lead to hyperuricemia. The interplay between OSA and hyperuricemia highlights the systemic nature of sleep apnea, affecting various biochemical pathways and contributing to the risk of developing other urate-related conditions.

One epidemiological study of 1101 volunteers was conducted to examine an association between OSA and uric acid [26]. OSA was determined by polysomnography, and those with OSA showed significantly higher levels of uric acid compared to the control group after adjusting for confounding factors. One meta-analysis also reported that individuals with OSA had higher serum uric acid levels compared to the control group [27]. Moreover, serum uric acid was associated with all-cause mortality and cardiovascular mortality in individuals with OSA which was determined using the NHANES screening questionnaire [28]. A bidirectional two-sample Mendelian randomization study was conducted using data from publicly available genome-wide association study summary statistics [29]. Genetically predicted OSA was significantly associated with increased serum uric acid levels after adjusting for confounders. This genetic link offers a clearer insight into the biological mechanisms that may predispose individuals to both conditions simultaneously. Clinical interventions, particularly the use of Continuous Positive Airway Pressure (CPAP) therapy, have shown promising results in managing both OSA and associated hyperuricemia. CPAP treatment has been documented to significantly reduce both the urinary excretion of uric acid and serum levels, potentially reversing the adverse metabolic effects induced by OSA-induced hypoxia [30,31]. These results from epidemiological studies, genetic studies, and therapeutic trials indicate evidence of a strong causal link between OSA and elevated uric acid. Our study results showing higher levels of uric acid in participants with a high risk of OSA and hyperuricemia as an independent risk factor for OSA are consistent with findings from previous studies. One intriguing finding of our study was the positive correlation between uric acid levels and STOP-Bang scores. This result was corroborated by a previous epidemiological study, which reported that serum uric acid levels correlated with OSA severity through measures such as an apnea-hypopnea index, desaturation index, and minimum saturation [26].

Socioeconomic status significantly influences the risk and severity of OSA, as evidenced by multiple studies across different populations [32]. A survey was conducted to identify the prevalence of OSA and its risk factors in the South Australian population. Regional area of residence, low education level, and low household income were significantly associated with a high risk of OSA (STOP-Bang score ≥ 3) [33]. A population-based study including 2162 participants in Switzerland also showed that lower occupation and education level were associated with a higher apnea-hypopnea index and oxygen desaturation index [34]. Moreover, a correlation exists between lower socioeconomic status and heightened disease severity in individuals with OSA, leading to increased healthcare expenses and an elevated risk of cardiovascular disease [35]. Further corroborating these findings, additional research has shown that lower socioeconomic status not only predisposes individuals to greater risks of developing OSA but also leads to increased healthcare expenditures and higher cardiovascular risks, due to the compounding effects of OSA on overall health [36]. This relationship is particularly notable as OSA exacerbates other comorbid conditions, which are more prevalent and often less managed in populations with lower income and education levels. Recognizing the influence of socioeconomic factors on OSA prevalence and severity, further studies are essential to explore how elements like education, income, and regional disparities impact the diagnosis, management, and outcomes of OSA, particularly in settings like Korea, where specific socio-economic factors might influence health outcomes differently.

One strength of this study is its large population number. A stratified sampling method was applied, such that the KNHANES can be described as representative of the Korean population. This is a distinctive feature in comparison to other epidemiological studies reporting on the relationship between OSA and uric acid. There are also several limitations in this study. First, high OSA risk was defined using a screening questionnaire (STOP-Bang), which is not the definitive diagnostic method for OSA [37]. The diagnostic accuracy of the screening questionnaire for OSA was analyzed in meta-analyses. The STOP-Bang questionnaire showed superior sensitivity (≥90%) and lower specificity (42%) compared to other screening questionnaires for OSA, possibly leading to the overestimation of the prevalence of OSA [38,39]. Second, the KNHANES involves a cross-sectional study design. Therefore, we could not examine longitudinal outcomes such as prognosis and mortality. Merging the dataset with longitudinal cohort data should be sought to evaluate the long-term effect of hyperuricemia in patients with a risk of OSA. Lastly, some questions included in the STOP-Bang questionnaire were self-reported, except blood pressure, age, neck circumference, and gender. Therefore, it is likely that the patient’s subjective criteria were followed. The assessment of snoring and fatigue, for example, may be relatively inaccurate compared to clinical assessment.

## 5. Conclusions

This study assessed the association of serum uric acid levels with the risk of OSA in the general population. A substantial proportion of the population are at high risk of OSA. Serum uric acid levels were significantly higher in those with a high risk of OSA and correlated with STOP-Bang scores. Further, hyperuricemia was independently associated with a high risk of OSA after adjusting for confounding factors. Considering the substantial portion of the population at high risk for OSA, as identified by STOP-Bang scores, and the significant correlations with hyperuricemia, further longitudinal studies are warranted. Future research should confirm OSA using definitive diagnostic methods and focus on the longitudinal outcomes of treating hyperuricemia, with an emphasis on sleep monitoring within the context of OSA management. It should also examine whether interventions that target uric acid levels can mitigate the progression or severity of OSA. Additionally, exploring the genetic predispositions to both conditions could elucidate underlying mechanisms further and help in identifying individuals at risk more effectively. This comprehensive approach could lead to more personalized and effective management strategies, potentially reducing the public health burden associated with OSA and related metabolic disturbances. Through continued investigation and a deeper understanding of these complex interactions, we can better address the multifaceted challenges posed by OSA and hyperuricemia, improving patient outcomes and quality of life.

## Figures and Tables

**Table 1:** Baseline characteristics of study subjects.

Variables	Total (*n* = 11,981)	STOP-Bang (≤2) (*n* = 7409)	STOP-Bang (≥3) (*n* = 4572)	*p* Value
Age (Mean ± SD)	59.9 ± 11.7	57.7 ± 11.8	63.4 ± 10.6	<0.001
40–49	2853 (23.8)	2336 (31.5)	517 (11.3)	<0.001
50–59	3074 (25.7)	1909 (25.8)	1165 (25.5)	
60–69	3139 (26.2)	1709 (23.1)	1430 (31.3)	
≥70	2915 (24.3)	1455 (19.6)	1460 (31.9)	
Men (*n*, %)	5206 (43.5)	1961 (26.5)	3245 (71.0)	<0.001
Smoking history (%)				
Never	7143 (59.7)	5357 (72.4)	1786 (39.1)	<0.001
Former	3017 (25.2)	1196 (16.2)	1821 (39.9)	
Current	1807 (15.1)	849 (11.5)	958 (21.0)	
BMI (%)				
<18.5	314 (2.6)	256 (3.5)	58 (1.3)	<0.001
18.5–24.9	7170 (59.8)	5037 (68.0)	2133 (46.7)	
25.0–29.9	3843 (32.1)	1992 (26.9)	1851 (40.5)	
≥30	654 (5.5)	124 (1.7)	530 (11.6)	
Medical history				
Hypertension	4081 (34.1)	1186 (16.0)	2895 (63.3)	<0.001
Hyperlipidemia	3469 (29.0)	1648 (22.2)	1821 (39.8)	<0.001
Stroke	369 (3.1)	144 (1.9)	225 (4.9)	<0.001
Myocardial infarct or angina	524 (4.4)	193 (2.6)	331 (7.2)	<0.001
Diabetes	1700 (14.2)	708 (9.6)	992 (21.7)	<0.001
Chronic kidney disease	179 (1.5)	78 (1.1)	101 (2.2)	<0.001
Gout	286 (2.4)	81 (1.1)	205 (4.5)	<0.001
Household income				
Q1	2633 (22.1)	1430 (19.4)	1203 (26.4)	<0.001
Q2	3016 (25.3)	1813 (24.6)	1203 (26.4)	
Q3	3033 (25.4)	1965 (26.6)	1068 (23.4)	
Q4	3254 (27.3)	2171 (29.4)	1083 (23.8)	

SD, standard deviation; BMI, body mass index.

**Table 2:** Risk factors associated with high risk of OSA.

Variables	Univariate Analysis OR (95% CI)	*p* Value	Multivariate Analysis OR (95% CI)	*p* Value
Age				
40–49	Ref.		Ref.	
50–59	2.80 (2.44–3.20)	<0.001	4.71 (3.98–5.59)	<0.001
60–69	3.53 (3.10–4.02)	<0.001	5.47 (4.58–6.54)	<0.001
≥70	3.70 (3.23–4.23)	<0.001	6.61 (5.48–7.96)	<0.001
Men (*n*, %)	6.62 (6.02–7.27)	<0.001	10.25 (8.68–12.10)	<0.001
Smoking history (%)				
Never	Ref.		Ref.	
Former	4.50 (4.05–5.01)	<0.001	1.23 (1.04–1.46)	0.016
Current	3.34 (2.94–3.79)	<0.001	1.20 (0.98–1.46)	0.073
BMI (%)				
<18.5	Ref.		Ref.	
18.5–24.9	1.83 (1.27–2.62)	0.001	1.73 (1.17–2.56)	0.006
25.0–29.9	4.34 (3.01–6.25)	<0.001	3.98 (2.67–5.94)	<0.001
≥30	20.93 (13.88–31.57)	<0.001	53.80 (34.06–84.99)	<0.001
Medical history				
Hyperlipidemia	2.55 (2.31–2.81)	<0.001	2.50 (2.19–2.86)	<0.001
Stroke	2.71 (2.13–3.43)	<0.001	1.59 (1.19–2.12)	0.002
Myocardial infarct or angina	3.01 (2.40–3.79)	<0.001	1.44 (1.08–1.91)	0.012
Diabetes	2.64 (2.34–2.99)	<0.001	1.33 (1.14–1.55)	0.000
Chronic kidney disease	1.99 (1.34–2.96)	0.001	2.25 (1.40–3.61)	0.001
Gout	3.98 (2.98–5.31)	<0.001	1.56 (1.14–2.15)	0.006
Household income				
Q1	Ref.		Ref.	
Q2	0.80 (0.70–0.91)	0.001	0.87 (0.73–1.03)	0.111
Q3	0.66 (0.59–0.74)	<0.001	0.76 (0.65–0.89)	0.001
Q4	0.66 (0.59–0.74)	<0.001	0.76 (0.64–0.90)	0.002
Hyperuricemia	2.01 (1.78–2.27)	<0.001	1.30 (1.11–1.53)	0.001

BMI, body mass index.

**Table 3:** Patient characteristics before and after propensity score matching.

	Before Propensity Score Matching			After Propensity Score Matching		
Variables	Low OSA Risk (*n* = 7409)	High OSA Risk (*n* = 4572)	SMD	Low OSA Risk (*n* = 3408)	High OSA Risk (*n* = 3408)	SMD
Body mass index	23.4 ± 3.0	25.5 ± 3.6	2.061	24.6 ± 3.0	24.8 ± 3.5	0.178
Age	58.6 ± 12.2	63.6 ± 10.7	4.979	62.4 ± 12.5	62.6 ± 10.5	0.191
Neck circumference	33.8 ± 2.8	37.2 ± 3.2	3.374	35.8 ± 2.6	36.2 ± 2.9	0.397
Male gender (*n*, %)	1961 (26.5)	3245 (71.0)	−0.448	1903 (55.8)	2115 (62.1)	−0.062

OSA, obstructive sleep apnea; SMD, standardized mean difference.

**Table 4:** Logistic regression analysis to identify factors associated with high OSA risk in a matched cohort.

Variables	Univariate Analysis OR (95% CI)	*p* Value	Multivariate Analysis OR (95% CI)	*p* Value
Snoring	18.78 (15.58–22.63)	<0.001	86.99 (62.73–120.65)	<0.001
Tiredness	10.92 (9.56–12.47)	<0.001	63.97 (47.93–85.38)	<0.001
Observed apnea	29.79 (20.60–43.08)	<0.001	132.30 (80.13–218.45)	<0.001
Hypertension	6.79 (6.09–7.58)	<0.001	68.08 (51.50–89.98)	<0.001
Uric acid level	1.06 (1.02–1.09)	0.003	1.36 (1.28–1.44)	<0.001

OR, odds ratio; CI, confidence interval.

## Data Availability

All data are available from the database of Korean National Health and Nutrition Examination Survey (KNHANES: https://knhanes.kdca.go.kr/knhanes/eng/index.do, accessed on 18 October 2023). KNHANES allows access to all these data, without approval or cost, for any researcher who promises to follow the research ethics guidelines. Those seeking access to this article’s data can download them from the website after promising to follow the research ethics guidelines.
